# Specific ocular changes in TTR Met30-FAP after liver transplantation

**DOI:** 10.1186/1750-1172-10-S1-P64

**Published:** 2015-11-02

**Authors:** Natalia Ferreira, David Dias, Carmen Vilas-Boas, Isabel Fonseca, Teresa Coelho

**Affiliations:** 1 Ophthalmology Department - Centro Hospitalar do Porto – Hospital Santo António (HSA), Porto, Portugal; 2 Centro Hospitalar do Porto, Unidade Corino de Andrade, Porto, Portugal; 3 Neurology Department – Centro Hospitalar do Porto – HSA, Unidade Corino de Andrade, Porto, Portugal

## Purpose

To report ocular manifestations in Portuguese TTR Met30 FAP patients submitted to liver transplant (LT).

## Methods

Retrospective observational consecutive case series of 78 LT recipients, observed by one ophthalmologist (FN) between January 2007 and July 2015. Demographic data, age at beginning of disease, period of evolution of disease, ophthalmological alterations and ocular surgeries (glaucoma surgery and vitrectomy) were evaluated. Patients were divided in three groups according to date of LT: less than 5 years (group 1); between 5 and 10 years (group 2) and more than 10 years (group 3).

## Results

Thirty-six patients were male. Twelve, 32 and 34 patients belong to group 1, 2 and 3 respectively. The mean age of patients was 41 years (group 1), 44 years (group 2) and 50 years (group 3). Symptoms and signs of dry eye were present in 33% (group1), 56% (group 2) and 62% (group 3) of patients. Amyloid deposits on pupillary border were observed in 16%, 27% and 41% of eyes according to each group. Scalloped pupil was present in 16%, 25% and 36% of cases, respectively. Amyloid vitreous opacities could be seen in 33%, 40% and 59% of eyes. Vitrectomy and glaucoma surgery was performed in 19 and 24 eyes of group 3, respectively.

## Conclusion

The prevalence of specific ocular changes increase with time after LT. The need of vitrectomy or glaucoma surgery is common 10 years after LT. Regular ophthalmological evaluation of FAP patients submitted to LT is important to accurately treat sight-threatening manifestations.

